# Endovascular Treatment of Giant Splenic Artery Aneurysm

**DOI:** 10.1155/2012/964093

**Published:** 2012-12-17

**Authors:** Adenauer Marinho de Oliveira Góes Junior, Amanda Silva de Oliveira Góes, Paloma Cals de Albuquerque, Renato Menezes Palácios, Simone de Campos Vieira Abib

**Affiliations:** ^1^Department of Medical Habilities, Federal University of Pará (UFPA), Belém, PA, Brazil; ^2^Department of Surgery, Federal University of São Paulo (UNIFESP), São Paulo, SP, Brazil

## Abstract

*Introduction*. Visceral artery aneurysms are uncommon. Among them, splenic artery is the most common (46–60%). Most splenic artery aneurysms are asymptomatic and diagnosed incidentally, but its rupture, potentially fatal, occurs in up to 8% of cases. *Presentation of Case*. A female patient, 64 years old, diagnosed with a giant aneurysm of the splenic artery (approximately 6.5 cm in diameter) was successfully submitted to endovascular treatment by stent graft implantation. *Discussion*. Symptomatic aneurysms and those larger than 2 cm represent some of the main indications for intervention. The treatment may be by laparotomy, laparoscopy, or endovascular techniques. Among the various endovascular methods discussed in this paper, there is stent graft implantation, a method still few reported in the literature. *Conclusion*. Although some authors still consider the endovascular approach as an exception to the treatment of SAA, in major specialized centers these techniques have been consolidated as the preferred choice, reserving the surgical approach in cases where this cannot be used. For being a less aggressive approach, it offers an opportunity of treatment to patients considered “high risk” for surgical treatment by laparotomy/laparoscopy.

## 1. Introduction

Visceral artery aneurysms (VAAs) are uncommon. Among them, splenic artery is the most common (46–60%), followed by hepatic artery (20%) and superior mesenteric artery (5-6%) [1–6].

 Seventy-five percent of VAAs are asymptomatic. The most common symptom is pain in the upper left quadrant of the abdomen or in the epigastrium, radiating to left shoulder, nausea, and vomiting. [1, 2, 7, 8]. The rupture occurs in 3% to 8% of cases, is manifested by hypovolemic shock, and is potentially fatal [1, 2, 4, 7, 8].

 The splenic artery aneurysms can be approached by laparotomy, laparoscopy, or endovascular techniques. The endovascular option, less invasive, has less morbidity and faster postoperative recovery [1–4, 8].

 Among the various endovascular techniques, covered stent implantation has been little reported in the literature. The authors present a case of splenic artery aneurysm treated by this method.

## 2. Presentation of Case

A female patient, 64 years old, controlled hypertension, and 2 previous pregnancies, presented as main complaint episodes of mild pain in the epigastrium and left hypochondrium, evolving for about 12 months. Physical examination of the abdomen was nonspecific, with ill-defined pain on palpation of the mesogastrium and left hypochondrium.

**Figure 1:**
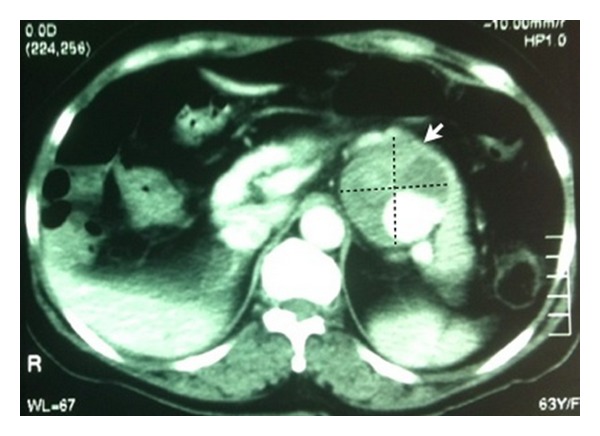
Axial section of CT of the abdomen with intravenous contrast. The arrow indicates the underlying lesion to the pancreas (splenic artery aneurysm).

 In the hospital of origin she was submitted to computerized tomography (CT) revealing a mixed density lesion with heterogeneous enhancement after injection of intravenous contrast (IV), relating to the pancreatic tail, measuring 6.5 cm in diameter (Figure 1).

 Considering the appearance of the lesion on CT, the diagnosis of splenic artery aneurysm was suggested and the patient referred to our institution to be submitted to an angiography for further diagnostic evaluation and possible endovascular treatment.

**Figure 2:**
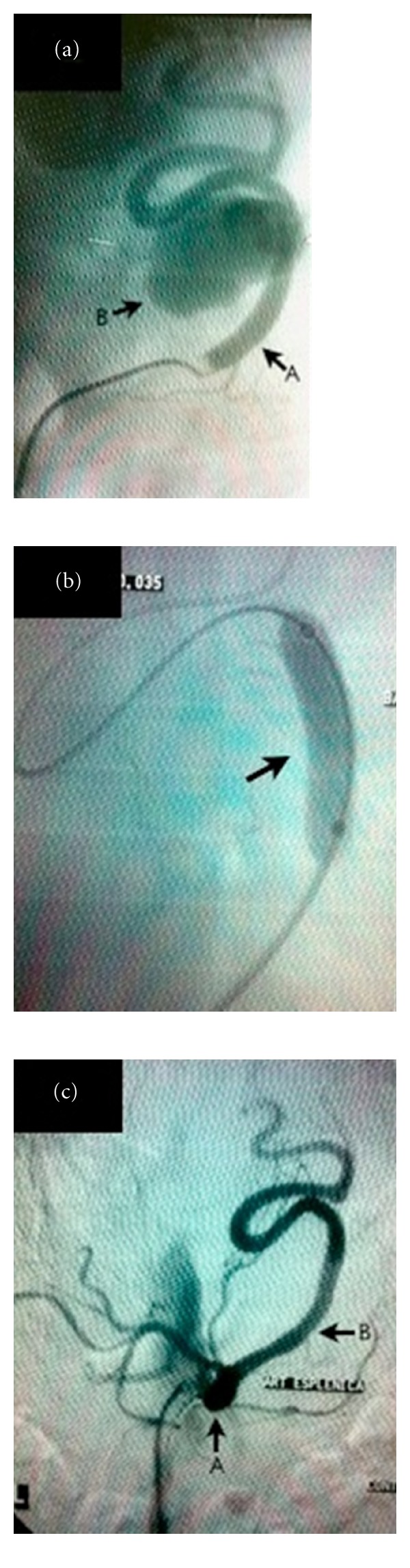
(a) Preoperative angiography. A: splenic artery, B: saccular aneurysm. (b) 6 × 40 mm angioplasty balloon inflated to stent accommodation. (c) Control angiography showing absence of perfusion of the aneurysmal sac. A: celiac trunk, B: splenic artery.

 The examination, performed by puncture of the right common femoral artery, with angiographic series of the abdominal aorta, celiac trunk, and splenic artery identified a patent and tortuous splenic artery, presenting a voluminous saccular aneurysm, with large neck, in the transition between their proximal and medium segments (Figure 2(a)).

Endovascular treatment was indicated and executed in a second moment.

 The procedure was carried out under sedation and local anesthesia. Initially a puncture of the right common femoral artery was performed and a 5F sheath was installed. The celiac trunk and then splenic artery were catheterized with a C2 5F catheter. With the aid of road map, a hydrophilic guide wire 0.035′′ × 260 cm was positioned beyond the aneurysm, until a terminal branch of the splenic artery.

The angiographic catheter was advanced over the hydrophilic guide wire, which was replaced by an extra-rigid one, that once positioned, and allowed to exchange the catheter and the 5F sheath for an angiographic 9F sheath, which had its distal extremity positioned at the origin of the splenic artery. The patient received heparin intravenously.

 After a new road map, a 6 × 50 mm Viabahn stent graft (Gore) was released on the topography of the aneurysm neck. An 6 × 40 mm angioplasty balloon was used for expansion and stent accommodation (Figure 2(b)).

 Intraoperative control arteriography showed patency of the celiac trunk and splenic artery and absence of aneurysm opacification (Figure 2(c)).

A manual compression of the puncture site was performed for 30 minutes and then compressive dressing. Immediately after the procedure the patient received 300 mg of clopidogrel.

 In the following days the patient received clopidogrel 75 mg/day and acetylsalicylic acid 200 mg/day and progressed without complaints or complications until discharge from hospital on the second postoperative day (POD). 

**Figure 3:**
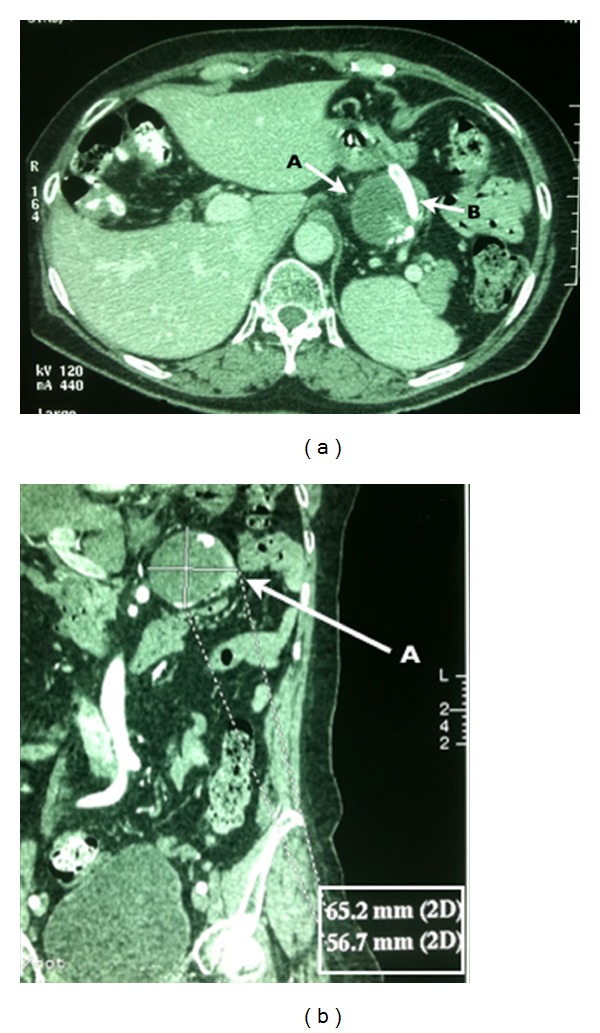
Computed tomography of the abdomen with intravenous contrast (postoperative control). (a) Axial section: A: thrombosed aneurysm sac, B: flow of contrast through the implanted stent. (b) Coronal section: A: thrombosed aneurysm sac, highlighted at the bottom of the figure, the measures of the aneurysm.

 On 30° POD, the patient remained without complaints or signs of complications. A CT with intravenous contrast was made on the 32nd postoperative day and confirmed thrombosis of the aneurysm sac and patency of the implanted stent, without evidence of splenic ischemia (Figure 3).

 The patient continues to be followed, with clopidogrel prescription until the sixth postoperative month and permanent use of aspirin. The episodes of abdominal pain ceased since the intervention.

## 3. Discussion

Visceral artery aneurysms (VAAs) have an incidence of 0.1% to 2% in the general population [2]. The most affected vessel is the splenic artery, corresponding to 60% of all VAAs [1–4].

 The splenic artery originates from the celiac trunk and is responsible for vascularization of the spleen, pancreas, and the stomach [7]. The first case of splenic artery aneurysm (SAA) was described in 1770 by Beaussier [4]. 

 The disease is more prevalent in women (75–87%) [1, 4, 5, 7] between 50 and 79 years old [1, 4]. The main factors associated to SAA are atherosclerotic disease, portal hypertension, cirrhosis, and multiple pregnancies. Other etiologies include fibromuscular dysplasia, neurofibromatosis, Ehlers-Danlos syndrome, infections, congenital abnormalities, pancreatitis, septic emboli, hypertension, vasculitis, trauma, and liver transplantation [1, 3–9].

 Most aneurysms are smaller than 2 cm and located in the middle or distal vessel segment [1, 3, 5–7]. They are multiple in 20% [5, 7] and the saccular morphology is predominant [1, 3–7].

 Seventy-five percent are asymptomatic [1, 2, 5, 7, 8]. The most common symptoms are pain in the left upper abdomen that may radiate to the shoulder, nausea, and vomiting [1, 5–7]. Rupture is the most feared complication and occurs in 2% to 10% of cases; courses with acute abdominal pain, hypotension, and hypovolemic shock. Risk factors for rupture are diameter larger than 2 cm, pregnancy, atherosclerosis, arterial hypertension, intra-abdominal inflammation process, trauma, collagenosis, fibromuscular dysplasia, cirrhosis, and liver transplantation, due to reduced resistance in the portal vein leading to increased blood flow in the splenic artery [1, 2, 4, 6–9]. Mortality from rupture ranges from 36% [7, 8] to 70% [1, 3, 4]. Other complications include the formation of an arteriovenous fistula with consequent portal hypertension [7].

 Usually, SAAs are incidental findings. Due to the development of radiological methods, the frequency of the diagnosis is increasing [2, 3, 5–7]. In simple abdomen radiograph, it may present itself as calcification in the left upper quadrant. The diagnosis can be confirmed by CT, magnetic resonance imaging (MRI), Doppler ultrasound, or arteriography [1, 6, 7].

 The high-resolution CT and arteriography can confirm the diagnosis of small SAA, but this last one locates more precisely, defines the size and the presence of other aneurysms, and still offers the possibility of therapeutic intervention. Generally, an arteriography is performed for planning of surgery intervention [2, 7, 9].

 The intervention is indicated for symptomatic aneurysms despite its diameter, to those larger than 2 cm, expanding aneurysms, women of childbearing age, pregnant women before the third quarter, in the presence of portal hypertension and if there is possibility of liver transplantation [2–4, 6, 7, 10]. Aneurysms with a diameter of 1-2 cm should be monitored every six months by imaging methods [7].

 Treatment can be performed by laparotomy, laparoscopy, or by endovascular technique. The surgical procedure consists of ligation of the aneurysm neck or aneurysmectomy with or without reconstruction of the artery and may require splenectomy.

 The mortality of the endovascular treatment varies between 0.5 and 5% in elective situations and can reach 25% in emergency procedures [2]. Succeeds in 70–95% of cases and in many specialized centers are considered the treatment of choice for VAAs with compatible anatomy and noncontraindications to the method.

 The endovascular options include transcatheter embolization with coils [1, 3–11], associated or not to stent implantation [1, 3–11], percutaneous injection of coils [3, 6–8] or thrombin [3] within the aneurysm, and the use of covered stents [1, 3, 4, 7, 8, 11]. The endovascular approach of SAA is an efficient alternative, less aggressive, and with lower morbidity when compared to conventional surgical treatment [1, 3, 5–11].

 Transcatheter embolization with coil is indicated in cases with favorable anatomy, for narrow-necked saccular aneurysms and in the presence of adequate collateral circulation, once there is distal embolization risk [5, 8].

 This technique has been successfull in 85% of cases [7, 8]. Some authors consider it the procedure of choice for treatment of SAA, especially if located in the distal third of the vessel, near the hilum [1, 3], is inadvisable in giant aneurysms, because of the possibility of inefficient occlusion of the aneurysm sac, the chance of an intense inflammatory process, and the risk of coil migration, which may cause infarction visceral. Other complications include the presence of aneurysmal rupture during endovascular manipulation and abscess formation [4, 6–8, 10]. The presence of metal coils can also affect postoperative interpretation of radiologic images [11].

 The injection of thrombin directly into the aneurysm, through percutaneous puncture, is a possibility when transcatheter embolization is not feasible or was not successful [3].

 Covered stents represent an option for patients with the proximal and distal splenic artery preserved [2]. Aneurysms located at proximal or medial-thirds of the vessel, as in this case, represent the best indications for this technique [1, 4, 5]. Aneurysms of the distal third of the vessel (mainly located in the splenic hilum) should not be treated by this method. Marked arterial tortuosity and an unfavorable angulation of the celiac trunk with the aorta are factors that may hinder the progression of the stent to the suitable site for release [1, 10, 11]. Balloon-mounted covered stents release more accurately, but the self-expanding ones progress better by tortuous arteries, a reason why it was chosen in this case [11].

 The use of endoprosthesis reduces the incidence of ischemic complications caused by embolization of coils. Even so, splenic infarction is the main complication of this approach, although it occurs in up to 40%, normally does not require splenectomy [2, 5].

 The use of covered stents is considered a good alternative and promising technique, but so far few cases have been reported [1, 3, 7, 8, 10]. Complications are related to stent migration and arterial occlusion; the first one can be prevented by choosing endoprosthesis with appropriate diameter, while the occlusion, even if it occurs, is usually slow and gradual, allowing the formation of adequate collateral circulation [11].

 In addition to specific complications of each endovascular method described above, postembolization syndrome, contrast nephropathy, hematoma at the puncture site, and transient elevation of pancreatic enzymes may occur. The paralytic ileus, infectious complications, bleeding, and acute pancreatitis, most common in conventional surgical treatment, rarely occur.

 After endovascular treatment, periodic CT or Doppler ultrasound follow-up is recommended [2, 3]. 

## 4. Conclusion 

 Although some authors still consider the endovascular approach as an exception to the treatment of SAA [8], in major specialized centers these techniques have been consolidated as the preferred choice [3, 7], reserving the surgical approach in cases where this cannot be used.

 For being a less aggressive approach, it offers an opportunity of treatment to patients considered “high risk” for surgical treatment by laparotomy/laparoscopy.
